# Alternative Splicing Dysregulation in Retinitis Pigmentosa: Pathogenic Mechanisms and Therapeutic Opportunities

**DOI:** 10.3390/biom15111624

**Published:** 2025-11-19

**Authors:** Yuxin Jiang, Xuyu Liu, Jie Fu, Yican Wu, Shanshan Yu, Kai Yao

**Affiliations:** 1 Institute of Visual Neuroscience and Stem Cell Engineering, Wuhan University of Science and Technology, Wuhan 430065, China; 2 College of Life Sciences and Health, Wuhan University of Science and Technology, Wuhan 430065, China

**Keywords:** alternative splicing, retinitis pigmentosa, gene regulation, pathological mechanisms, therapeutic strategies

## Abstract

Retinitis pigmentosa (RP) represents a genetically heterogeneous group of inherited retinal dystrophies characterized by progressive photoreceptor degeneration and irreversible vision loss. Among the diverse pathogenic mechanisms, dysregulation of alternative splicing has emerged as a pivotal driver, particularly in RP cases caused by mutations in splicing factors or cis-regulatory elements. Alternative splicing governs transcript diversity and fine-tunes gene expression, with more than 95% of human multi-exon genes undergoing this process. Disruption of precise splicing patterns in the retina—an organ with exceptionally high transcriptional complexity—leads to widespread mis-splicing of photoreceptor-specific genes, triggering retinal dysfunction and cell death. This review synthesizes current understanding of alternative splicing-related mechanisms in RP, integrating molecular insights from splicing-factor mutations, retina-specific splice isoforms, and their downstream cellular consequences. We also evaluate therapeutic strategies targeting splicing dysregulation, including antisense oligonucleotides (ASOs), modified U1 snRNA, spliceosome-mediated RNA trans-splicing (SMaRT), and genome editing, emphasizing translational potential and clinical challenges. Finally, we highlight key research gaps and propose future directions for splicing-centered precision medicine in RP.

## 1. The Pathogenesis of Retinitis Pigmentosa

**Figure 1:**
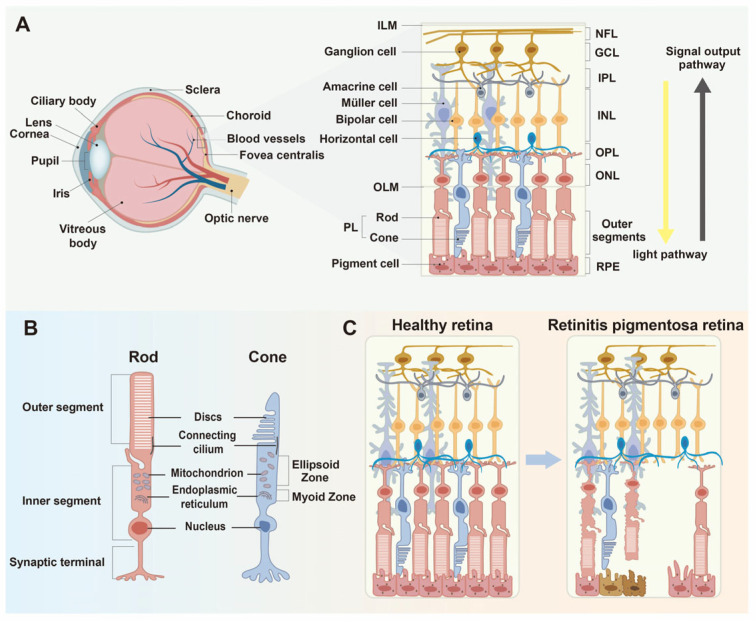
Retinal structure and pathological changes in retinitis pigmentosa (RP). (**A**) Anatomical overview of the human eye showing the cornea, lens, vitreous body, optic nerve, and layered organization of the retina. The neural retina is organized into sequential layers, including the inner limiting membrane (ILM), nerve fiber layer (NFL), ganglion cell layer (GCL), inner plexiform layer (IPL), inner nuclear layer (INL), outer plexiform layer (OPL), outer nuclear layer (ONL), outer limiting membrane (OLM), and photoreceptor layer (PL). (**B**) Structural schematics of rod and cone photoreceptors showing their outer and inner segments, mitochondria-rich ellipsoid zone, and synaptic terminals that transmit light signals. (**C**) Comparative illustration of a normal retina and one affected by RP. RP pathology begins with progressive rod degeneration, followed by secondary cone loss and accompanying retinal pigment epithelium (RPE) atrophy, ultimately leading to photoreceptor disorganization and vision impairment.

The retina is a delicate central nervous system (CNS) tissue layer situated in the posterior segment of the eye, serving as the primary site for phototransduction and visual signal processing [1]. It is embryologically derived from the neuroectoderm, a specialized region of the early embryonic ectoderm that gives rise to both the central and peripheral nervous systems [2]. The neural retina terminates posteriorly at the tips of the photoreceptor outer segments, whereas the retinal pigment epithelium (RPE) forms a single layer of pigmented cells immediately posterior to the retina. Although both the neural retina and RPE originate from the neuroectoderm, the RPE is not considered a neural layer of the retina but rather a distinct monolayer that plays a crucial supportive role in photoreceptor homeostasis. The retina consists of multiple organized layers, including the nerve fiber layer (NFL), ganglion cell layer (GCL), inner plexiform layer (IPL), inner nuclear layer (INL), outer plexiform layer (OPL), and outer nuclear layer (ONL) [3] (Figure 1A). The RPE provides structural support, metabolic nourishment, and clearance of photoreceptor outer-segment debris, maintaining retinal homeostasis [4]. Within the retina, the primary sensory cells are photoreceptors, comprising rods and cones. Cones mediate color discrimination and high-acuity vision under photopic (bright light) conditions, whereas rods are specialized for scotopic (low-light) vision [5] (Figure 1B).

Retinitis Pigmentosa (RP) is an inherited retinal dystrophy characterized by the progressive degeneration of photoreceptor cells and atrophy of the RPE. Its etiology is genetically heterogeneous, involving mutations in numerous genes expressed in photoreceptors and RPE, and it represents one of the leading causes of irreversible blindness worldwide [6]. The typical clinical manifestations of RP include night blindness, gradual loss of peripheral vision, and eventual central visual loss in the late stages of the disease [7]. These symptoms correspond to a sequential degeneration of rods followed by secondary cone loss and RPE dysfunction [8] (Figure 1C). Funduscopic examination reveals pathognomonic bone-spicule-like pigmentation, attenuation of retinal vessels, and waxy pallor of the optic disks [9]. Beyond primary photoreceptor degeneration, secondary complications frequently arise, including cystoid macular edema (CME) and posterior subcapsular cataract (PSC), which further compromise visual acuity and quality of life [10]. To date, over 100 RP-associated genes have been cataloged (https://retnet.org/summaries#b-diseases (accessed on 1 September 2025)), encompassing diverse biological processes such as phototransduction, RNA splicing, ciliary transport, lipid metabolism, and cytoskeletal regulation. This genetic diversity underlies the broad spectrum of clinical phenotypes observed in RP.

Heterogeneity is a hallmark of RP, with variability observed in the age at onset, rate of disease progression, and degree of visual impairment [11]. Following Mendelian inheritance patterns, RP can be classified into three major genetic forms: autosomal dominant (adRP, 30–40%), autosomal recessive (arRP, 50–60%), and X-linked (XLRP, 5–15%) [12]. In addition, several atypical inheritance modes have been identified, including X-linked dominant, mitochondrial, and digenic variants [13]. Clinically, RP can also be categorized as syndromic or non-syndromic. Non-syndromic RP represents the predominant type, manifesting solely with ocular lesions [14]. By contrast, syndromic RP is associated with systemic abnormalities such as Usher syndrome (USH)—a ciliopathy characterized by retinitis pigmentosa and sensorineural hearing loss, Bardet–Biedl syndrome (BBS)—a multisystem ciliopathy featuring retinal degeneration, obesity, and polydactyly and rarer genetic disorders including PHARC (polyneuropathy, hearing loss, ataxia, retinitis pigmentosa, and cataract syndrome), PCARP (photoreceptor ciliopathy and retinal pigmentation), and Oliver–McFarlane syndrome (OMCS) [15,16].

## 2. The Mechanism of Alternative Splicing

**Figure 2:**
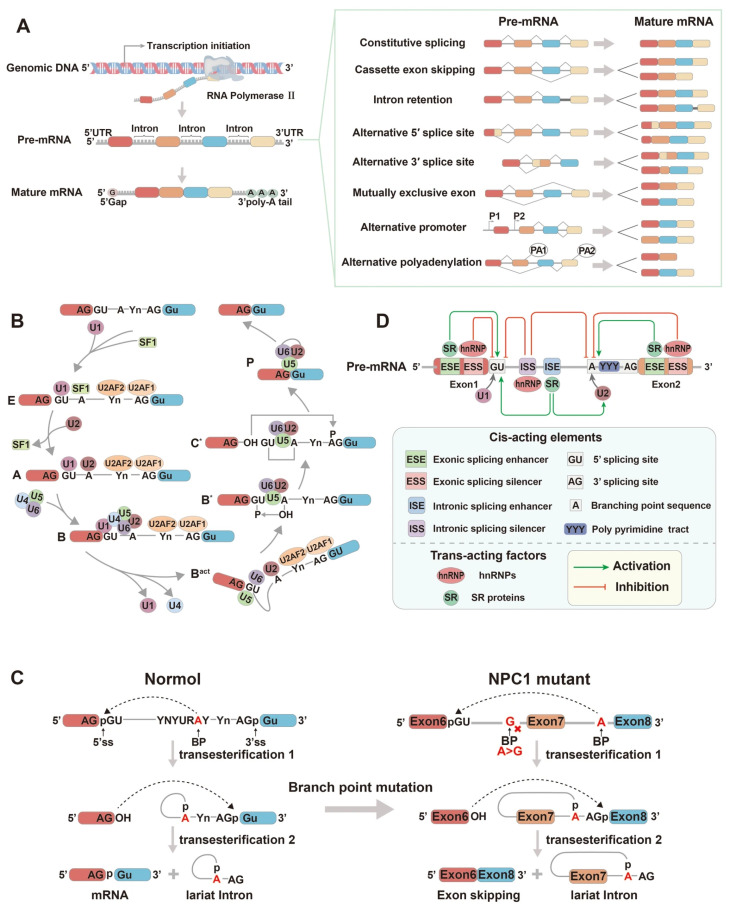
Overview of alternative splicing mechanisms and regulation. (**A**) Major forms of alternative splicing. Depending on regulatory signals, a single pre-mRNA can be processed through distinct patterns—such as exon skipping, intron retention, alternative 5′ or 3′ splice sites, mutually exclusive exons, alternative promoters, or alternative polyadenylation—to generate multiple mature mRNA isoforms. (**B**) Stepwise assembly and catalytic cycle of the spliceosome. The process involves the sequential recruitment of U1, U2, U4/U6, and U5 snRNPs and associated factors to form the E, A, B, and C complexes. U1 recognizes the 5′ splice site, while U2 binds to the branch-point sequence (BPS). Subsequent conformational rearrangements activate the spliceosome for the two-step transesterification reactions, leading to exon ligation and intron excision as a lariat RNA. (**C**) Defects in branch-point recognition and lariat formation. Under normal conditions (left), accurate pairing between the 5′ splice site and the branch-point adenosine allows proper lariat formation and exon ligation through two transesterification steps. In contrast, mutations within the branch-point region, such as *NPC1* c.882-28A>G, disrupt 5′ lariat formation and hinder exon recognition, resulting in exon 7 skipping and abnormal splicing (right). This defect illustrates how errors in 5′-end lariat formation compromise transcript integrity and contribute to disease pathogenesis. (**D**) Regulatory networks of alternative splicing. Splicing decisions are determined by the interplay between cis-acting elements—including exonic/intronic splicing enhancers (ESEs/ISEs) and silencers (ESSs/ISSs)—and trans-acting factors such as SR proteins (activators) and hnRNPs (inhibitors). These elements and proteins coordinate splice-site selection to maintain the balance between isoform diversity and splicing fidelity.

RNA splicing is a fundamental step in gene expression in which introns are removed from pre-mRNA and exons are joined to form mature transcripts. Depending on the regulatory context, splicing can proceed via constitutive or alternative pathways [17]. Constitutive splicing eliminates all introns in a fixed order to generate a single canonical mRNA, whereas alternative splicing selectively employs different splice sites to produce multiple mRNA isoforms from the same precursor, thereby expanding transcript and protein diversity [18]. Seven major forms of alternative splicing have been identified: exon skipping, alternative 5′ splice sites (A5SS), alternative 3′ splice sites (A3SS), mutually exclusive exons (MXE), intron retention (IR), alternative promoters, and alternative polyadenylation [19] (Figure 2A). More than 95% of human multi-exon genes undergo alternative splicing, greatly enhancing the proteomic complexity of higher eukaryotes [20].

Splicing is executed by the spliceosome, a highly dynamic ribonucleoprotein (RNP) complex composed of small nuclear RNAs (snRNAs) and associated proteins [21]. It recognizes conserved intronic elements—the 5′ splice site (5′ ss), 3′ splice site (3′ ss), branch point sequence (BPS), and polypyrimidine tract (PPT)—and catalyzes intron excision and exon ligation [22]. Two major spliceosomal systems operate in eukaryotes: the U2-dependent (major) and U12-dependent (minor) pathways. The U2-dependent spliceosome (U1, U2, U4, U5, U6 snRNPs) removes the vast majority of introns, while the U12-dependent complex (U11, U12, U4atac, U5, U6atac) processes a rare class of U12-type introns [23]. Each snRNP contains a specific snRNA and a group of associated protein factors, such as Sm or LSm proteins, which collectively contribute to the accuracy and efficiency of the splicing process [24].

The formation of the spliceosome is a highly orchestrated and dynamic process characterized by extensive conformational and compositional rearrangements that facilitate the sequential assembly of small nuclear ribonucleoproteins (snRNPs) together with numerous non-snRNP factors, ultimately resulting in the formation of catalytically active spliceosomal subcomplexes [25] (Figure 2B). Initially, U1 snRNP recognizes the GU dinucleotide at the 5′ ss through base pairing between its U1 snRNA and the pre-mRNA, an interaction stabilized by the U1C protein. Subsequently, splicing factor 1 (SF1) and the U2 auxiliary factors (U2AF2 and U2AF1) bind to the BPS, PPT, and AG dinucleotide at the 3′ ss, establishing the early (E) complex. Upon ATP hydrolysis, U2 snRNP displaces SF1 and associates with the branch-point to form the A complex [26]. The U4/U6·U5 tri-snRNP complex then joins the A complex in an ATP-dependent manner, forming the catalytically inactive B complex. This complex undergoes major rearrangements and conformational transitions to yield the pre-catalytic Bact complex, during which U1 and U4 snRNP dissociate. The activated spliceosome (B*) then catalyzes the first trans-esterification reaction [27,28]. The second trans-esterification occurs within the C* complex, ligating the two exons to produce the post-splicing (P) complex, which contains the joined exons [29]. Finally, the excised intron is released as a lariat structure, completing the splicing cycle. The mature mRNA is exported for translation, while snRNPs are recycled for subsequent rounds of splicing [30].

During the two-step catalytic cycle, intron excision proceeds through the transient formation of a lariat intermediate. In the first transesterification, the 2′-hydroxyl group of the branch-point adenosine attacks the 5′ splice site, forming a 2′–5′ phosphodiester bond; in the second step, the 3′-hydroxyl of the 5′ exon joins the 3′ splice site to ligate exons, releasing the intron as a lariat RNA. Notably, accurate lariat formation at the 5′ end of the intron is essential for the successful completion of the first trans-esterification step. The efficiency of DBR1-mediated debranching is strongly influenced by the identity of the branch-point nucleotide—preferentially adenosine—and the surrounding cis-regulatory motifs [31]. Mutations that disrupt these elements, such as *NPC1* c.882-28A>G, can impair lariat formation and resolution, resulting in exon skipping or activation of cryptic splice sites [32] (Figure 2C).

Building upon the precisely orchestrated catalytic steps of pre-mRNA splicing, the regulation of alternative splicing adds an additional layer of complexity that ensures transcript diversity while preserving splicing fidelity. A vast array of mRNA isoforms is thereby produced through the intricate coordination of cis-acting elements and trans-acting factors [33] (Figure 2D). Cis-acting elements are short RNA sequences within pre-mRNA that include canonical splice sites, branch-point sequences, and splicing enhancers or silencers located in either exonic or intronic regions. These elements influence splice-site selection by interacting with specific regulatory proteins [34]. Trans-acting factors fall primarily into two major families: serine/arginine-rich (SR) proteins and heterogeneous nuclear ribonucleoproteins (hnRNPs). SR proteins, characterized by their arginine–serine dipeptide motifs, bind to exonic splicing enhancers (ESEs) via RNA-recognition motifs, facilitating the recruitment of U1 and U2 snRNPs and enhancing splice-site recognition [35]. Conversely, hnRNPs preferentially associate with exonic or intronic splicing silencers (ESSs/ISSs), antagonizing splice-site selection and promoting exon skipping or intron retention [36]. Moreover, tissue-specific regulators, such as RBM20 and PTBP1, coordinate splicing events in a cell-type-dependent manner—for instance, modulating cardiac exon usage to maintain cardiomyocyte function [37]. An imbalance between cis- and trans-regulatory components can result in aberrant splicing, compromising both transcript diversity and fidelity. Additionally, mutations in core spliceosomal proteins or snRNA components lead to defective mRNA processing, contributing to a broad spectrum of human diseases, including retinitis pigmentosa, cancer, and immune dysregulation [38].

## 3. The Mechanisms of Alternative Splicing in Retinitis Pigmentosa

In recent years, the study of alternative splicing has become a rapidly expanding field in retinal molecular genetics. Accumulating evidence indicates that AS plays a decisive role in shaping the functional transcriptome of the retina, an organ characterized by exceptionally high transcriptional complexity and metabolic demand. Disruptions in precise splicing patterns lead to the production of aberrant mRNA isoforms, functional inactivation of critical retinal genes, and subsequent photoreceptor degeneration. Rather than representing a secondary effect of mutation, splicing dysregulation itself constitutes an active pathogenic driver that modulates disease onset, severity, and phenotypic variability in RP. For example, mutations in the *RPGR* gene are a major cause of X-linked RP (XLRP), and aberrant splicing of this gene has been strongly correlated with disease severity and variable expressivity among patients [39]. A novel splicing-site mutation, *RPGR* c.619+1G>C, produces aberrant transcripts and defective protein expression, illustrating the direct pathogenic potential of splicing errors [40]. Similarly, *PRPF8* mutations—encoding a core spliceosomal component—cause defective splice-site selection and decreased generation of retina-specific transcripts [41]. These discoveries have redefined RP as a prototypical “spliceosomopathy,” in which global perturbations of RNA processing manifest as tissue-restricted retinal degeneration.

Irregularities in alternative splicing not only disrupt gene expression but also contribute to the wide spectrum of RP clinical phenotypes. For instance, a deep intronic *RPGR* mutation (c.779−5T>G) significantly reduces splicing efficiency, producing distinct transcript isoforms that correlate with variable disease severity [42]. This highlights that differential AS regulation can partially explain the clinical heterogeneity of RP, bridging molecular mechanisms with patient outcomes. Therapeutically, the reversibility of AS provides a unique entry point for intervention. By manipulating splicing choices through RNA-based therapeutics, normal gene expression patterns can potentially be restored. For example, antisense oligonucleotides (ASOs) designed to modulate *RPGR* splicing have successfully improved transcript fidelity and protein expression in preclinical models [40]. These results underscore the dual significance of AS in RP—as both a key pathogenic mechanism and a promising therapeutic target.

### 3.1. Splicing Factors in the Mechanisms of Retinitis Pigmentosa

**Figure 3:**
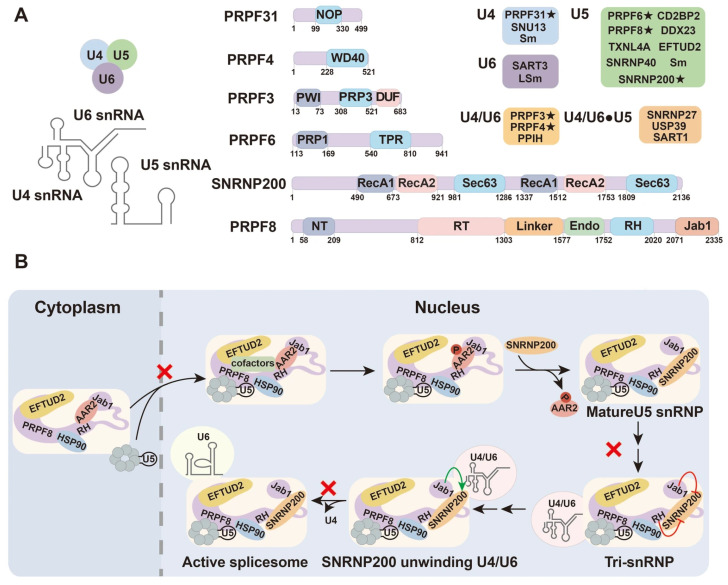
Composition of the U4/U6·U5 tri-snRNP complex and its pathogenic relevance in RP. (**A**) Schematic representation of the U4, U5, and U6 snRNAs and their associated protein components within the tri-snRNP complex, along with domain structures of representative spliceosomal proteins. Proteins marked with “★” denote splicing factors implicated in retinitis pigmentosa (adapted from the NCBI database, https://www.ncbi.nlm.nih.gov/). (**B**) Splicing factor mutations and their functional consequences in retinitis pigmentosa. Overview of RP-associated splicing factor genes, including *PRPF3*, *PRPF8*, *PRPF31*, *SNRNP200*, *PRPF6*, and *PRPF4*, and their positions within the spliceosomal complex. Mutations in these genes disrupt RNA–protein interactions, lariat excision, or tri-snRNP stability, resulting in defective mRNA processing and photoreceptor degeneration. The schematic highlights distinct mechanistic nodes (U4/U6·U5 tri-snRNP assembly, U2 binding, RNA helicase function) that are perturbed in RP. The red “×” symbol highlights the processes affected by the mutated PRPF.

In adRP, numerous mutations have been identified in genes encoding core spliceosomal components, including *PRPF3*, *PRPF4*, *PRPF6*, *PRPF8*, *PRPF31*, *SNRNP200* (*Brr2*), *PAP-1* (*RP9*), *DHX38*, and *CWC27* [43]. With the exception of *RP9*, *DHX38*, and *CWC27*, most of these genes encode proteins that assemble into the U4/U6·U5 tri-snRNP complex. Despite their ubiquitous expression across human tissues, mutations in these genes selectively impair retinal cells, implying that photoreceptors are uniquely sensitive to disturbances in splicing homeostasis [44]. These splicing factors exert their functions through specialized structural domains that mediate the precise assembly and dynamic rearrangement of spliceosomal complexes (Figure 3A). Mutations in PRPF family proteins can disrupt the nuclear–cytoplasmic trafficking or catalytic activation of spliceosomal subunits, leading to mis-splicing of retinal transcripts, proteotoxic stress, and ultimately photoreceptor apoptosis. This dysfunction contributes to the progressive degeneration characteristic of RP (Figure 3B).

#### 3.1.1. U4 snRNP Specific Protein-PRPF31

PRPF31 directly binds U4 snRNA and facilitates the interaction between U4/U6 di-snRNP and U5 snRNP, thereby promoting the formation of the U4/U6·U5 tri-snRNP complex essential for pre-mRNA splicing [45]. Knockdown of *PRPF31* disrupts U4/U6·U5 association, hindering tri-snRNP assembly and splicing fidelity [46]. *PRPF31* is the most frequently mutated splicing factor gene in autosomal dominant retinitis pigmentosa (adRP), accounting for approximately 8.9% of all cases and corresponding to RP type 11 (RP11). Its mutations include missense, nonsense, and large deletions, often exhibiting incomplete penetrance. Genetic modifiers such as CNOT3 and eQTLs at chromosome 14q21–23 have been proposed to influence this variability [47,48]. Functional studies in zebrafish and mammalian retinal models demonstrate that *Prpf31* deficiency leads to mitotic arrest and DNA damage in retinal progenitor cells (RPCs), triggering p53-mediated apoptosis [49].

**Figure 4:**
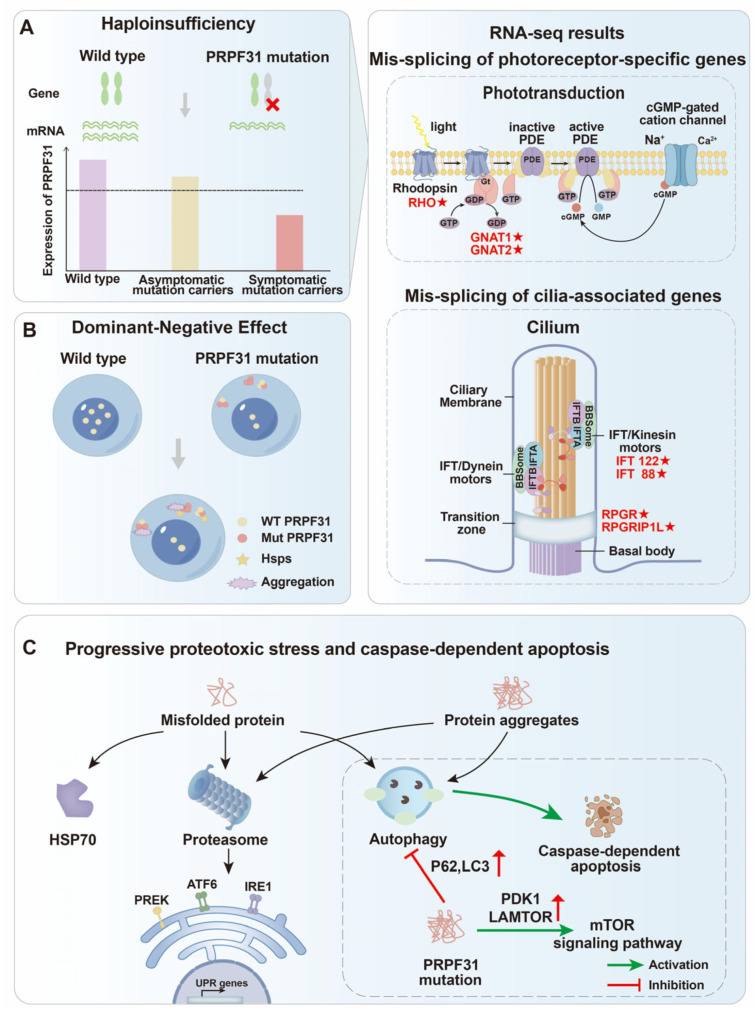
Photoreceptor physiology and consequences of splicing defects in RP. (**A**) Haploinsufficiency: Heterozygous mutations in *PRPF31* lead to reduced gene expression and insufficient spliceosomal activity, resulting in aberrant splicing of photoreceptor-specific genes (e.g., rhodopsin signaling components) and cilia-associated genes (e.g., *IFT* and *RPGR*). Regions marked with red “★” indicate aberrant splicing of genes caused by PRPF31 mutations. The red “×” denotes the *PRPF31* mutation. The outer segment cGMP-gated cation channel conducts primarily Na^+^ (~90%) and minor Ca^2+^ currents. (**B**) Dominant-negative effect: Mutant PRPF31 proteins abnormally interact with wild-type counterparts, disrupting their localization and forming insoluble cytoplasmic aggregates. These aggregates induce stress responses involving molecular chaperones such as heat-shock proteins (HSPs). (**C**) Proteotoxic stress and caspase-dependent apoptosis: Accumulation of misfolded PRPF31 proteins and protein aggregates triggers proteotoxic stress, activating the unfolded protein response (UPR), autophagy, and caspase-dependent apoptotic pathways, ultimately causing photoreceptor cell death. Red arrows indicate upregulation.

RNA-seq analyses from retinal organoids (ROs) and *Prpf31* heterozygous mouse models show that reduced *Prpf31* expression causes extensive exon skipping and intron retention in key genes related to phototransduction (*RHO*, *GNAT1*, *GNAT2*) and splicing regulation (*PRPF3*, *PRPF4*, *PRPF8*), resulting in impaired photoreceptor function [50]. Moreover, pathogenic *PRPF31* mutations, such as c.1115_1125del11 and c.522_527+10del, induce aberrant splicing of ciliogenesis- and adhesion-related genes (*IFT122*, *IFT88*, *RPGRIP1*) in retinal pigment epithelium (RPE) and photoreceptors, leading to defective ciliary structures and increased cellular stress [51] (Figure 4A).

In the *Prpf31^p.A216P/+^* mouse model, both haploinsufficiency and dominant-negative mechanisms operate simultaneously: mutant proteins form insoluble cytoplasmic aggregates that sequester wild-type PRPF31, disrupt its nuclear function, and activate compensatory chaperones such as HSP70 family proteins [52] (Figure 4B). However, these compensatory responses are insufficient to prevent retinal degeneration, emphasizing the delicate stoichiometric balance required for splicing fidelity. In some cases, structural deletions (e.g., c.1115_1125del11) prevent proper incorporation of the mutant PRPF31 into the spliceosome, resulting in cytoplasmic accumulation of misfolded protein aggregates and activation of autophagy–lysosome pathways. Pharmacologic activation of autophagy by rapamycin has been shown to clear PRPF31 aggregates and partially restore RPE cell viability, suggesting a mechanistic link between splicing defects, proteostasis, and retinal cell survival [53] (Figure 4C). To date, at least 177 unique *PRPF31* mutations have been identified (https://www.hgmd.cf.ac.uk/ac/index.php (accessed on 1 September 2025)), encompassing frameshift, splice-site, nonsense, and large deletions (Table 1). Collectively, these data highlight PRPF31 as a pivotal regulator of spliceosome integrity, whose dosage sensitivity renders retinal cells particularly vulnerable to its perturbation.

#### 3.1.2. U4/U6 snRNP Specific Proteins-PRPF3 and PRPF4

The PRPF3 protein serves as an essential bridging component within the spliceosome, mediating the interaction between U4/U6 di-snRNPs and U5 snRNP, thus ensuring the stability of the tri-snRNP complex [77]. Mutations in *PRPF3* cause autosomal dominant RP type 18 (RP18), which typically presents in adolescence or early adulthood and progresses slowly compared to other RP forms [78]. Among these, the Thr494Met variant in exon 11 is the most frequent and impairs phosphorylation-dependent PRPF3 self-association and binding to PRPF4 and U4/U6 snRNA, leading to defective complex assembly and reduced splicing efficiency [79].

Similarly, PRPF4 plays a critical role in tri-snRNP biogenesis through its WD40 repeat domain, which scaffolds protein–protein interactions with PRPF3 and PPIH [80]. Two heterozygous mutations—c.-114_-97del and c.C944T (p.Pro315Leu)—have been identified in RP families, with the former reducing *PRPF4* promoter activity and protein levels, and the latter triggering compensatory upregulation of other spliceosomal components [81]. The p.R192H variant acts as a loss-of-function allele that disrupts the PRPF3–PRPF4 binding, preventing proper tri-snRNP incorporation and highlighting the structural interdependence of U4/U6-associated proteins in splicing fidelity [82]. These findings highlight the essential function of PRPF4 in spliceosome assembly and illustrate the unique molecular pathways through which mutations in *PRPF4* lead to RP.

#### 3.1.3. U5 snRNP Specific Proteins—PRPF8, PRPF6, and SNRNP200

The *PRPF8* gene encodes the largest and most conserved catalytic component of the spliceosome, which orchestrates both assembly and catalytic activation of the U4/U6·U5 tri-snRNP complex [83]. Mutations in *PRPF8* underlie autosomal-dominant RP type 13 (RP13) and are frequently located in the Jab1/MPN domain at the C-terminus [84]. This domain regulates the helicase Brr2 (SNRNP200), ensuring proper unwinding of the U4/U6 RNA duplex during spliceosome activation [85]. Pathogenic variants such as p.H2309P disrupt this regulation, leading to either premature activation or stalled catalysis. As a result, defective spliceosome remodeling and mis-selection of 5′ splice sites occur, causing retina-specific splicing errors and photoreceptor dysfunction [41]. Furthermore, *PRPF8* mutations promote cytoplasmic mislocalization and excessive binding to HSP90/R2TP chaperone complexes, which impairs nuclear import of the protein and reduces interaction with U5 snRNA. This mislocalization produces a dual insult—loss of splicing function in the nucleus and gain of toxic aggregation in the cytoplasm—culminating in apoptosis of photoreceptor cells [86]. In addition, mutant *Prpf8* mice show disrupted circadian rhythms and reduced rhythmic gene expression in retinal tissue, illustrating that spliceosomal dysfunction extends beyond transcript processing to broader cellular homeostasis [87]. Under hypoxic stress, certain *PRPF8* mutations (e.g., R2310K) enhance aberrant skipping of *ULK1* exons 22–23, suppressing mitophagy and aggravating retinal degeneration [88]. These results collectively establish *PRPF8* as a hub connecting splicing regulation with cellular stress adaptation pathways.

PRPF6, another U5-specific protein, acts as a structural bridge linking the U5 and U4/U6 snRNPs [89]. The c.2185C>T (p.Arg729Trp) mutation co-segregates with adRP and causes aberrant accumulation of PRPF6 within nuclear Cajal bodies, leading to intron retention and inefficient spliceosome assembly [90]. Induced pluripotent stem cell (iPSC)-derived RPE models carrying this mutation exhibit compromised barrier function and ciliogenesis [91]. Importantly, combined dysregulation of *PRPF6* and *PRPF31* alters the expression of *TTLL3*, the only enzyme responsible for microtubule monoglycylation in human retina, linking splicing defects to microtubule instability and ciliary degeneration [92].

The helicase SNRNP200 (Brr2), a core U5 component, contains two distinct helicase cassettes—*N*-terminal catalytic and C-terminal regulatory domains—that mediate the ATP-dependent unwinding of the U4/U6 duplex [93]. Mutations such as Ser1087Leu and Arg1090Leu within the Sec63-like domain reduce helicase activity and promote cryptic splice-site usage without altering tri-snRNP incorporation [94]. iCLIP analyses have revealed that these mutations impair RNA binding and duplex unwinding efficiency, thereby disrupting spliceosome activation [95]. A recently identified mutation, c.C6088T (p.Arg2030Cys), acts via a dominant-negative mechanism; its overexpression in zebrafish embryos causes retinal malformations and photoreceptor death, providing strong in vivo evidence for mutation-driven toxicity [96].

#### 3.1.4. Non-snRNP Splicing Regulatory Factors: PAP-1 (RP9), DHX38, and CWC27

Beyond core snRNP components, several non-snRNP splicing regulators are also implicated in RP pathogenesis. *PAP*-*1* (*RP9*) encodes a splicing cofactor that interacts with Pim-1 kinase and U2AF35, modulating pre-mRNA splicing efficiency [97]. Pathogenic variants such as D170G reduce its phosphorylation and disrupt co-localization with SC35 nuclear speckles, impairing retina-specific transcript processing [98]. CRISPR/Cas9-mediated knockout and point-mutant (H129L) models have shown decreased proliferation and migration of photoreceptor cells, accompanied by defective splicing of *FSCN2* but not *BBS2*, suggesting selective regulatory control [99]. Interestingly, mutations in *PRPF31* (e.g., c.544_618del75bp) can secondarily impair the assembly of the U4/U6·U5 tri-snRNP complex and disturb normal *PRPF31* function, thereby inducing aberrant splicing of downstream targets such as *RP9*. This cross-regulatory effect between core and non-core splicing factors highlights the interconnected nature of the spliceosomal network. Such abnormal splicing cascades promote photoreceptor apoptosis and contribute to RP pathogenesis [100]. These results indicate that RP9 acts as a fine-tuning factor, regulating subsets of transcripts critical for photoreceptor maintenance.

*DHX38* (PRP16), a DEAH-box helicase, functions during the transition between spliceosomal complexes B and C [101]. A missense mutation (c.995G>A; p.Gly332Asp) identified in Pakistani families causes early-onset RP and macular hole formation [102]. Functional analyses revealed that the mutant protein selectively impairs splicing of *RHO* and promotes the use of cryptic splice sites, reducing splicing fidelity for suboptimal substrates. Zebrafish models further demonstrated that *dhx38* deficiency induces R-loop-associated DNA damage and p53-dependent apoptosis of RPCs [103]. Thus, *DHX38* not only regulates canonical splicing but also safeguards genomic stability during retinal development.

*CWC27* encodes a peptidyl-prolyl isomerase-like protein that forms a heterodimer with *CWC22*, bridging the exon junction complex (EJC) to the spliceosome [104]. Although the *N*-terminal PPIase domain lacks enzymatic activity, it mediates critical protein–protein interactions necessary for EJC deposition and mRNA export. Loss-of-function mutations in *CWC27* cause syndromic or non-syndromic RP with varying systemic defects. The *Cwc27^K338fs^*^/^*^K338fs^* mouse model exhibits extensive intron retention, alternative splice-site selection, and ER stress–induced photoreceptor degeneration [105,106]. Together, these non-snRNP regulators demonstrate that perturbations in peripheral spliceosomal networks are sufficient to elicit photoreceptor-specific vulnerability.

### 3.2. Disease Modeling Using iPSC-Derived Retinal Pigment Epithelium and Organoids

Although studies in animal and cell models have elucidated critical aspects of spliceosomal gene dysfunction, the molecular mechanisms by which these mutations selectively impair human photoreceptors remain only partially understood. The majority of current data are derived from non-retinal or non-human systems, which cannot fully recapitulate the metabolic and splicing complexity of the human retina. Recent advances in patient-derived induced pluripotent stem cell (iPSC) technology have made it possible to generate retinal pigment epithelium (RPE) and retinal organoids (ROs) that faithfully mimic human retinal architecture and physiology. These iPSC-based models provide an unprecedented opportunity to dissect the cellular consequences of splicing-factor mutations, validate disease-associated pathways, and evaluate therapeutic interventions in a patient-specific context.

In iPSC-derived RPE and ROs from *PRPF31*-mutated RP11 patients, Buskin et al. reported extensive alternative splicing defects in genes involved in splicing and ciliogenesis, leading to disrupted apical–basal polarity, reduced barrier integrity, impaired phagocytosis of outer segments, and shortened or bulbous primary cilia. CRISPR/Cas9 correction of the *PRPF31* mutation restored normal splicing, RPE architecture, and ciliary morphology, providing direct proof-of-concept for gene repair in splicing-factor RP [51].

Similarly, Liang et al. demonstrated that iPSC-RPE cells harboring *PRPF6* mutations show defective cell polarity and calcium signaling abnormalities [91], whereas Atkinson et al. found that the PRPF8 p.H2309P mutation disrupts Brr2-mediated helicase function, resulting in aberrant nuclear speckle organization and photoreceptor degeneration [41]. Rodrigues et al. further confirmed that *PRPF31/PRPF8*-mutant ROs recapitulate cilium assembly defects and altered nuclear architecture in retinal cells [107]. Moreover, stem-cell-based replacement strategies using iPSC-derived RPE or retinal progenitor cells have entered early clinical testing. A systematic review of 21 trials reported that such transplantation improved visual acuity in ~49% of RP eyes with acceptable safety [108].

Collectively, these studies highlight iPSC-derived retinal models as a powerful platform for mechanistic dissection and preclinical evaluation of splicing-targeted therapies in RP.

### 3.3. The Mechanism of Cis-Regulatory Element Mutations in Retinitis Pigmentosa

In addition to mutations in spliceosomal proteins, pathogenic variants within cis-regulatory elements critically influence splicing accuracy in RP. These include canonical splice-site disruptions, branch-point alterations, polypyrimidine tract defects, and deep intronic variants that activate cryptic exons. Such mutations can result in exon skipping, intron retention, or pseudoexon inclusion []. For instance, an 18-bp deletion in intron 15 of *PDE6B* (c.1921-20_1921-3del) abolishes the canonical acceptor site, deleting six amino acids in the catalytic domain and leading to arRP [109]. Likewise, the *RPE65* c.1430A>G variant creates a novel splice site and generates aberrant transcripts that cause dominant RP [110]. The severe early-onset phenotype associated with *RHO* c.620T>G mutation further illustrates how a single nucleotide change can establish an aberrant splice acceptor and disrupt rhodopsin localization in photoreceptors [111].

Recent studies have emphasized the significance of deep intronic mutations—variants located far from exon-intron boundaries that activate pseudoexons or perturb splicing signal recognition [112]. A newly identified *CLRN1* c.254-643G>T mutation introduces a cryptic exon, producing a leaky transcript that explains the non-syndromic phenotype of RP without hearing loss [113]. Deep intronic variants in *USH2A* (e.g., c.7595-2144A>G, c.9959-2971C>T, c.8682-654C>G, and c.9055+389G>A) are increasingly recognized as pathogenic, supported by long-read sequencing validation [114,115,116]. These findings underscore that intronic splicing mutations constitute a major hidden cause of RP and highlight the diagnostic value of transcriptome-level analysis in cases unsolved by exome sequencing. A summary of selected gene-related mutations is provided in Table 2.

Collectively, the mechanisms described above establish a hierarchical model linking molecular splicing defects to RP pathogenesis. Mutations in core spliceosomal factors (PRPF31, PRPF8, SNRNP200) primarily compromise the catalytic efficiency and structural integrity of the spliceosome, leading to global splicing dysregulation and secondary transcriptomic stress. In contrast, mutations in non-core regulators (RP9, DHX38, CWC27) and cis-elements exert more localized effects, altering the splicing of specific photoreceptor or ciliogenesis genes. These two levels of dysfunction converge on common downstream pathways—protein aggregation, ciliopathy, ER stress, and apoptosis—culminating in retinal degeneration. From a translational perspective, this layered understanding offers a systematic framework to prioritize therapeutic targets. Core splicing factors represent upstream “master regulators” suitable for gene replacement, whereas cis-element and minor regulator mutations may be amenable to RNA-based correction using ASOs or U1 snRNA engineering. By integrating these mechanistic tiers, future research can establish a unified model linking genotype to splicing phenotype and ultimately to therapeutic response in RP.

A long-standing question in the field concerns why mutations in ubiquitously expressed spliceosomal components predominantly cause retinal degeneration. Several complementary hypotheses have been proposed to explain this tissue specificity.

First, photoreceptor cells have exceptionally high metabolic and biosynthetic demands: they continuously transduce light signals, propagate electrical activity, and renew the membrane disks of rod and cone outer segments. Consequently, photoreceptors operate at unusually high rates of transcription, translation, and protein trafficking [141]. Under this “high-load” state, photoreceptor function is exquisitely sensitive to the fidelity of pre-mRNA splicing—minor perturbations can destabilize cellular homeostasis and cause dysfunction. Indeed, normal retinal function critically depends on precise mRNA splicing and adequate gene expression; disturbances in these processes invariably trigger photoreceptor degeneration and cell death. Consequently, mutations in ubiquitously expressed spliceosomal genes (e.g., *PRPF31*) may be tolerated in most tissues but cause mis-splicing of photoreceptor maintenance transcripts in the metabolically demanding, splice-sensitive retina, ultimately leading to cellular degeneration. [50].

Second, the retina exhibits an unusually complex alternative-splicing (AS) landscape—over one quarter of retinal transcripts contain tissue-specific exons whose inclusion depends on finely tuned splicing kinetics; retina-enriched regulatory factors further shape photoreceptor-specific isoform expression and thereby amplify the consequences of spliceosomal dysfunction [142]. For example, Musashi proteins (MSI1 and MSI2) bind intronic sequences downstream of photoreceptor-specific exons to promote their inclusion and regulate the expression of photoreceptor-relevant proteins [143]. In addition, SRRM3 and SRRM4 are neuron-restricted splicing factors that orchestrate complex events, including microexon regulation. Loss of SRRM3 shortens and degenerates photoreceptor outer segments and impairs visual function in zebrafish models [144]. These findings indicate that retina-enriched splicing factors act cooperatively to ensure correct production of tissue-specific exons and isoforms.

Third, the retina has a distinctive reliance on oxidative phosphorylation. Emerging evidence shows that mitochondrial metabolism and NAD^+^ balance influence splicing efficiency, directly coupling energetic homeostasis to splicing fidelity [145]. Many spliceosomal activities (e.g., the U5 snRNP helicase) are ATP-dependent; energy shortfalls can slow splicing or increase splice-site errors. Conversely, cells can remodel splicing to accommodate energetic constraints—for instance, in renal epithelial cells, hypoxia induces specific splice variants that enhance mitochondrial output and protect thick ascending limb (TAL) cells from injury [146]. Analogous principles apply in the retina: mutations in the tricarboxylic-acid–cycle enzyme IDH3B, which participates in mitochondrial NAD^+^ production, cause homozygous non-syndromic retinitis pigmentosa without systemic metabolic defects [147].

Collectively, these factors render photoreceptors particularly sensitive to even minor disruptions in RNA processing. This framework helps explain the paradox that systemic mutations in spliceosomal genes cause selective retinal pathology while sparing most other tissues.

## 4. The Clinical Significance and Therapeutic Implications of Alternative Splicing in Retinitis Pigmentosa: Exploring Novel Treatment Strategies

The importance of alternative splicing in RP has garnered increasing attention, particularly in the context of developing novel therapeutic strategies. As mentioned previously, the pathogenesis of RP is closely associated with various gene mutations, including those affecting pre-mRNA splicing factors and cis-acting elements, which can lead to aberrant splicing events and ultimately result in RP. Therefore, correcting these splicing defects or restoring the normal expression of key genes has become a prominent focus in current research on RP. Currently, therapeutic strategies targeting alternative splicing abnormalities have evolved into a diversified landscape, primarily encompassing five major approaches: gene supplementation, antisense oligonucleotides (ASOs), U1 small nuclear RNA (U1snRNA) correction, spliceosome-mediated RNA trans-splicing (SMaRT), and gene editing technology. Although these modalities share the goal of restoring proper transcript processing, they differ substantially in mechanism, durability, and clinical readiness.

### 4.1. Gene Supplementation Therapy

**Figure 5:**
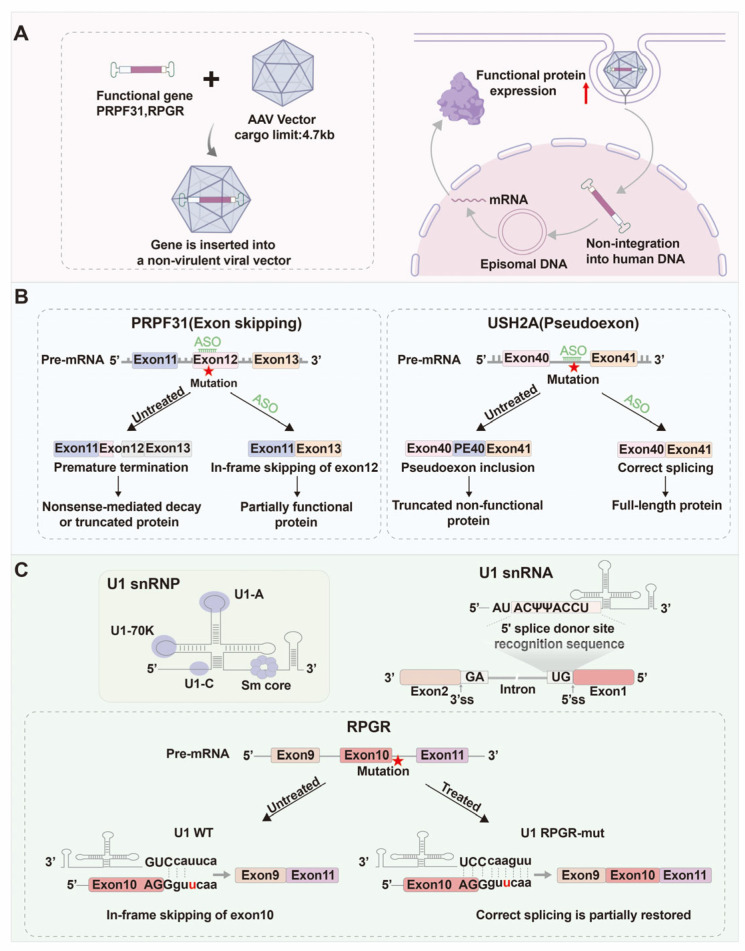
Therapeutic strategies targeting splicing defects in RP. (**A**) Gene supplementation therapy: Functional gene copies, such as *PRPF31* or *RPGR*, are packaged into adeno-associated virus (AAV) vectors with a cargo capacity of approximately 4.7 kb. Following subretinal delivery, episomal expression of the transgene restores normal protein levels and photoreceptor function without genomic integration. (**B**) Antisense oligonucleotide (ASO) therapy: ASOs are short, chemically modified single-stranded nucleotides designed to bind target pre-mRNAs and modulate aberrant splicing. Left panel: In *PRPF31*-associated RP11, an exon 12 mutation induces exon skipping, leading to nonsense-mediated decay or truncated protein products. ASO treatment promotes in-frame exon skipping and partial protein restoration. Right panel: In *USH2A*-related RP, intronic mutations generate pseudoexon 40 (PE40) inclusion, producing truncated usherin. ASO QR-421a corrects this defect by excluding the pseudoexon and reestablishing full-length transcript expression. (**C**) U1 snRNA engineering: U1 small nuclear RNA (U1 snRNA) recognizes the 5′ splice donor site during spliceosome assembly. Mutations at this site disrupt exon recognition and cause exon skipping. Top: The U1 snRNP complex consists of U1-70K, U1-A, and U1-C proteins assembled on the Sm core. Bottom: In *RPGR* mutations affecting exon 10 donor site, the wild-type U1 fails to recognize the mutated 5′ splice site, leading to exon 10 skipping. Engineered U1 snRNA (U1 *RPGR*-mut) restores correct pairing and partially rescues normal splicing. The red "★" symbols denote the mutation sites.

Gene supplementation therapy remains the most clinically advanced approach, particularly for recessive RP forms caused by loss-of-function mutations. This strategy employs adeno-associated virus (AAV) vectors to deliver functional gene copies to retinal cells, thereby compensating for defective alleles [148]. Mutations in *PRPF31* and *RPGR,* both key splicing-related genes, are prominent contributors to RP pathogenesis. Among the currently available treatments, Luxturna (Voretigene neparvovec-rzyl) represents a milestone, being the first FDA-approved therapy for *RPE65*-related RP and Leber congenital amaurosis (LCA12), in which AAV-mediated supplementation restored visual function in patients [149]. Beyond *RPE65*, preclinical and early clinical studies have explored supplementation for *PRPF31* and *RPGR*. AAV delivery of *PRPF31* rescued retinal structure and function in knockout mice, reducing apoptosis and gliosis [150], and improved phagocytic and ciliary activity in patient-derived RPE cells [151]. Similarly, restoration of *RPGR* expression using AAV5- or AAV2-based vectors (e.g., Botaretigene sparoparvovec, AGTC-501) showed favorable safety and promising improvements in retinal sensitivity, advancing toward phase III clinical trials [152,153] (Figure 5A). These results collectively confirm the therapeutic potential of gene replacement in RP.

However, critical challenges remain. The standard AAV capsid accommodates a maximum payload of ~4.7 kb, which limits application to large genes such as *USH2A* and *ABCA4* [154]. Overexpression-related cytotoxicity and variable transduction efficiency across retinal cell types further restrict therapeutic consistency. Moreover, gene supplementation is inherently unsuited for dominant-negative mutations, where pathogenic transcripts must be suppressed rather than supplemented. Thus, while gene therapy provides durable correction for recessive RP, its utility is narrowed by vector capacity and mutation type. In contrast, antisense oligonucleotide (ASO) therapy offers a more flexible and mutation-specific means to modulate RNA splicing directly. ASOs can correct exon skipping, induce exon inclusion, or suppress pseudoexons by base-pairing with target pre-mRNAs [155,156]. Importantly, ASOs do not rely on vector packaging, enabling rapid adaptation for individualized treatment. Nonetheless, their therapeutic effect is often transient and requires repeated intravitreal injections due to limited retinal penetration and turnover. Compared with gene supplementation, ASOs thus trade durability for precision and safety, providing an attractive but short-lived solution. Future strategies will likely integrate both platforms—using AAV for sustained expression of splicing-corrective elements and ASOs for fine-tuned, reversible modulation—to achieve an optimized balance between efficacy and safety.

### 4.2. Antisense Oligonucleotide (ASO) Therapy

In recent years, antisense oligonucleotide (ASO) therapy has rapidly advanced as a mutation-specific and RNA-level approach to correct aberrant splicing in inherited retinal diseases, including RP. ASOs are chemically modified, single-stranded nucleotides that hybridize to target pre-mRNA sequences to modulate splicing, induce exon skipping, or exclude pseudoexons [157]. Several preclinical and clinical studies have demonstrated their potential to restore functional transcripts and protein expression. For example, in *PRPF31*-related RP11, splice-switching ASOs targeting exon 12 successfully reestablished the open reading frame and increased *PRPF31* mRNA levels by 1.7-fold in patient-derived fibroblasts, achieving the functional threshold observed in asymptomatic carriers [66]. Similarly, the ASO QR-421a targeting *USH2A* exon 13 effectively promoted exon skipping and restored usherinΔexon13 expression, fully recovering retinal function in zebrafish and mouse models. The treatment progressed to phase 1/2 clinical evaluation (NCT03780257), where preliminary data confirmed its safety and durability [158,159].

Beyond single-exon targets, dual-exon and domain-oriented skipping strategies (e.g., *USH2A* exons 30–31 or 39–40) have expanded the therapeutic range by enabling in-frame deletions compatible with functional rescue [160]. Deep intronic pseudoexons, such as *USH2A* c.7595-2144A>G (PE40), were corrected through 2′-O-methyl phosphorothioate ASOs, restoring normal splicing in patient-derived cells [161]. Similar splicing modulation was achieved for *CLRN1* c.254-649T>G mutations in USH3, where ASO and CRISPR-based correction jointly reinstated proper splicing [162]. Notably, *CRB1*-associated RP exemplifies a condition in which exon-skipping-based correction may be required. The *CRB1* gene has a large coding sequence (~6.5 kb), which exceeds the packaging capacity of standard AAV vectors, rendering conventional gene supplementation approaches impractical. Recent clinical and therapeutic analyses have further confirmed that *CRB1* mutations represent a major challenge for AAV-based delivery systems, highlighting the potential of exon-skipping and RNA-level correction strategies as feasible alternatives to restore functional transcripts and rescue photoreceptor survival [163,164]. The action mechanisms of ASOs are diverse, capable of degrading pathogenic mRNA via an RNase H-dependent pathway, or regulate pre-mRNA splicing to induce exon skipping or pseudoexon exclusion [165]. The clinical translation of ASOs is further exemplified by sepofarsen (QR-110), targeting *CEP290* intronic variants, which improved visual function in LCA patients [157].

However, while ASO-based therapy demonstrates high mutation specificity and favorable safety, several limitations remain unresolved. First, the therapeutic effect is transient and requires repeated intravitreal injections, posing both patient burden and cumulative inflammation risk. Second, diffusion barriers and heterogeneous uptake across retinal layers can lead to variable efficacy. Third, each new mutation typically requires an independently optimized ASO design and regulatory path, limiting scalability. Compared to AAV gene supplementation, ASOs offer a rapid and reversible means of correction but lack long-term stability. To overcome these constraints, next-generation ASOs with vectorized or self-renewing expression and combinatorial regimens integrating ASO + CRISPR or ASO + gene supplementation are being actively explored to combine safety, precision, and durability (Figure 5B).

### 4.3. U1 snRNA Engineering

Aberrant recognition of the 5′ splice site (5′ ss) is another major pathogenic mechanism in RP, leading to defective spliceosome assembly and exon skipping [166]. In this context, U1 snRNA engineering has emerged as a promising RNA-based therapeutic strategy. U1 snRNA normally base-pairs with the 5′ ss to initiate spliceosome formation; thus, engineered U1 snRNA with complementary sequence adjustments can restore splicing fidelity at mutated site [167]. For instance, a mutation-specific U1 targeting *RHO* c.936G>A markedly reduced exon skipping and partially restored normal splicing in COS-7 cells and human retinal explants [168]. Similarly, customized U1 snRNA constructs corrected *RPGR* c.1245+A>T mutations, reducing aberrant splicing in patient-derived cells [169]. Moreover, antisense-modified U1 snRNA (U1_asRNA) successfully silenced pseudoexon 9a activation in *RPGR*, confirming its capacity to prevent cryptic splice site usage [139] (Figure 5C).

Nevertheless, the variability of outcomes across model systems highlights critical challenges in translation. U1 snRNA therapy is generally limited to single-point or donor-site mutations and depends on precise promoter and vector regulation to maintain physiological expression levels. Overexpression may saturate spliceosomal components and introduce off-target binding, while underexpression risks incomplete rescue. Despite these hurdles, U1 modification provides longer-lasting correction than ASOs, given its sustained vector-driven expression. Future optimization should focus on delivery standardization, model comparability, and safety validation to advance this approach toward clinical testing.

### 4.4. Spliceosome-Mediated RNA Trans-Splicing (SMaRT)

The spliceosome-mediated RNA trans-splicing (SMaRT) technique represents another RNA-level corrective strategy designed to reconstitute full-length, functional mRNA by replacing mutant segments with exogenous trans-splicing molecules (PTMs) [170]. By complementing specific pre-mRNA sequences, PTMs induce targeted trans-splicing between endogenous and corrective exons, thereby generating hybrid mRNAs encoding functional proteins [171]. This approach has shown success in several RP models. In *RHO*-mutant mice, AAV-delivered PTMs targeting intron 1 replaced exons 2–5, restoring proper protein localization and retinal morphology [172]. In *RPGR*-deficient models, trans-splicing repaired the ORF15 transcript, reinstating its guanine nucleotide exchange factor activity and maintaining photoreceptor homeostasis [173]. The mRNA trans-splicing recombinant technology (REVeRT) dual-AAV system further extended this concept to large genes, such as *ABCA4*, in Stargardt disease, effectively overcoming vector capacity limitations [174].

**Figure 6:**
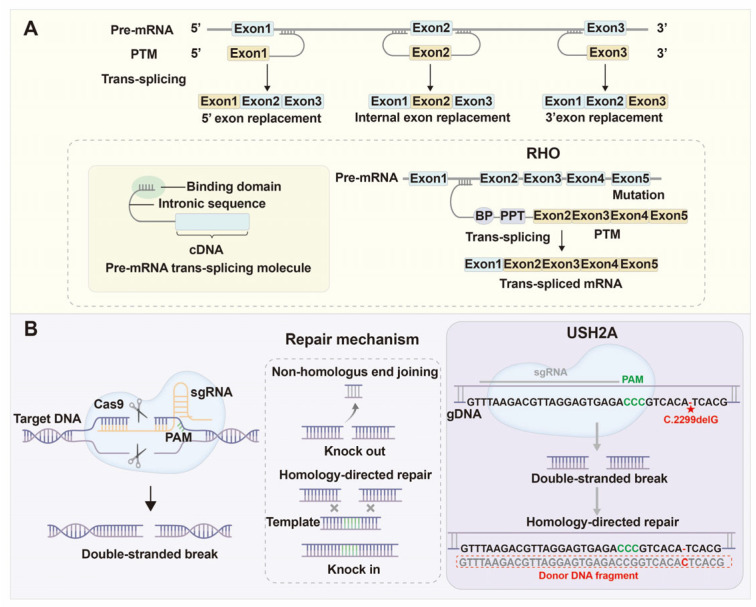
RNA- and DNA-based gene correction strategies for splicing-related RP. (**A**) Spliceosome-mediated RNA trans-splicing (SMaRT): The SMaRT approach repairs mutant pre-mRNAs by replacing defective exon segments with corrective RNA sequences through a spliceosome-dependent trans-splicing reaction. Top panels: Depending on the targeted region, pre-mRNA trans-splicing molecules (PTMs) can mediate 5′-, internal-, or 3′-exon replacement to generate hybrid mRNAs encoding full-length functional proteins. PTMs consist of a binding domain complementary to the target intronic sequence, an intronic spacer, and a corrective cDNA fragment. Bottom panel: In *RHO*-mutant models, AAV-delivered PTMs targeting intron 1 replace exons 2–5, producing trans-spliced mRNAs that restore correct rhodopsin localization and retinal morphology. (**B**) Genome editing technologies: CRISPR/Cas-based genome editing directly corrects pathogenic splicing mutations at the DNA level, enabling permanent gene repair. Left panels: The CRISPR/Cas9 complex uses a single-guide RNA (sgRNA) to direct Cas9 to a specific genomic sequence adjacent to a protospacer adjacent motif (PAM), generating a double-stranded DNA break. Repair occurs through non-homologous end joining (NHEJ), which introduces small insertions or deletions (knock-out), or homology-directed repair (HDR) using a donor template for precise sequence correction (knock-in). Right panel: In *USH2A*-related RP, CRISPR-mediated HDR corrects the c.2299delG mutation using an exogenous donor DNA fragment, restoring wild-type sequence integrity. The red ”★” indicates a gene mutation.

Despite its conceptual elegance, SMaRT faces persistent technical and safety barriers. Trans-splicing efficiency remains relatively low and prone to producing off-target chimeric transcripts [175]. Moreover, achieving optimal stoichiometry between PTMs and mutant pre-mRNA is experimentally challenging. The approach is best suited for diseases involving dominant-negative or splice-site mutations where direct exon replacement is feasible. While SMaRT offers a non-permanent, RNA-level repair pathway with theoretically reduced genomic risk, future development must prioritize enhancing efficiency and reproducibility before clinical translation (Figure 6A).

### 4.5. Genome Editing Technologies

Genome editing technologies provide the most direct avenue for permanent correction of splicing mutations at the DNA level [176]. The CRISPR/Cas9 system has enabled precise excision of pathogenic pseudoexons or correction of specific splice-disrupting mutations. Dual-sgRNA approaches successfully deleted intronic insertions in *CLRN1*, restoring wild-type transcript levels [162]. Correction of *PRPF31* (c.1115_1125del11) mutations in patient-derived iPSCs reinstated normal protein expression and ameliorated ciliopathy and polarity defects in RPE cells, demonstrating the functional impact of splicing repair [51]. Similarly, exon 13 mutations of *USH2A* were corrected via enhanced CRISPR (eDCas9) systems, achieving efficient splicing restoration [177]. In *RPGR*-related RP, excision of the ORF15 repetitive region via CRISPR/Cas9 restored reading frame and partial protein expression in rd9 mice, attenuating retinal degeneration [178].

Translational progress is exemplified by the EDIT-101 clinical trial, which uses SaCas9 delivered by AAV to remove intronic IVS26 mutations in *CEP290* responsible for LCA10. Interim Phase I/II results indicate encouraging safety and measurable visual improvement [179]. Additionally, base editors (CBE/ABE) and prime editors have expanded the scope of single-nucleotide correction without double-strand breaks, successfully rescuing *Pde6b*-related RP phenotypes in mice [180,181].

Despite its transformative potential, genome editing faces several unresolved controversies. The efficiency of homology-directed repair (HDR) remains extremely low in post-mitotic photoreceptors, and the risk of off-target mutations or unintended large deletions persists [182]. AAV delivery capacity limits multiplexed editing, while immune responses to bacterial Cas proteins may complicate repeated dosing. Moreover, ethical and regulatory concerns over in vivo genome editing necessitate rigorous safety validation. Nevertheless, genome editing offers unmatched durability and precision, and advances in high-fidelity Cas variants, non-viral delivery systems, and prime-editing fidelity optimization are steadily improving feasibility for retinal gene therapy (Figure 6B). To facilitate an integrated overview of ongoing therapeutic developments, we have summarized major strategies targeting alternative splicing abnormalities in retinitis pigmentosa, including their representative disease models, clinical progress, major findings, and associated limitations (Table 3).

Collectively, splicing-correction therapeutics in RP reflect a dynamic continuum of RNA- and DNA-level interventions. ASOs provide rapid, individualized correction with established safety; U1 snRNA and SMaRT extend correction duration through vectorized RNA engineering; and CRISPR-based approaches promise permanent repair at the genomic level. However, none of these modalities alone fully overcome the intertwined challenges of delivery, efficiency, and durability. The next generation of therapies will likely employ hybrid strategies—combining transient ASO modulation with durable vectorized or genome-editing systems—to balance precision and persistence. Cross-platform integration, coupled with AI-assisted optimization of splicing correction sites, may eventually enable patient-specific, lifelong treatments for RP.

## 5. Conclusions

Alternative splicing (AS) dysregulation has emerged as a central mechanism underlying the molecular and clinical heterogeneity of RP. Mutations in spliceosomal components and cis-regulatory elements perturb retinal transcript fidelity, leading to mis-splicing of photoreceptor- and cilium-associated genes, proteostasis imbalance, and progressive photoreceptor degeneration. Despite the retina’s high splicing complexity and cellular specialization, RP exemplifies how systemic spliceosomal dysfunction can produce tissue-specific pathology, underscoring the retina as a sensitive indicator of global RNA-processing disturbances.

From a mechanistic standpoint, RP-associated splicing defects converge on two interrelated processes: (1) Loss of splicing fidelity, driven by mutations in *PRPF31*, *PRPF8*, or *SNRNP200*, which disrupt tri-snRNP assembly and alter the kinetics of exon recognition. (2) Aberrant cis-element usage, including cryptic or deep intronic splice-site activation, which expands transcriptomic diversity at the expense of functional precision. These mechanisms jointly reshape the retinal transcriptome, impair ciliogenesis, and activate stress and apoptotic cascades. Consequently, therapeutic strategies must account for both global spliceosomal integrity and locus-specific correction.

Recent findings have also emphasized the intersection between splicing regulation, metabolic signaling, and noncoding RNA-mediated control in ocular and neurodegenerative contexts. Retinal cells exhibit extraordinarily high metabolic activity, and disruptions in metabolic homeostasis can exacerbate splicing defects. For instance, insulin–AKT–SRSF5–PKCβ and m^6^A–FTO–Runx1t1 axes dynamically reshape exon selection in response to glucose and lipid fluctuations, thereby coupling metabolic signaling with transcriptome adaptation. Moreover, accumulating evidence underscores the role of noncoding RNAs in splicing regulation. Long noncoding RNAs (lncRNAs) such as *MALAT1*, *MIAT*, and *NEAT1* modulate SR-protein localization within nuclear speckles, influencing exon inclusion and photoreceptor-specific transcript balance. In diabetic and degenerative retinal disease models, *MALAT1* was shown to coordinate metabolic and splicing pathways by regulating SRSF2 phosphorylation and PTBP1 expression. MicroRNAs (miR-124, miR-135b) also fine-tune splicing networks by targeting splicing factors or epigenetic modulators. Together, these data reveal that the retina functions as an integrative model where metabolic and ncRNA-driven regulation converge on splicing fidelity, providing mechanistic insight into tissue-specific vulnerability and therapeutic modulation.

Therapeutically, the expanding toolkit of splicing-correction technologies represents a continuum from RNA-level modulation to genomic repair. ASO-based therapies provide mutation-specific, reversible correction and are clinically advanced but require frequent administration. U1 snRNA modification extends correction durability through vectorized RNA delivery, though it remains mutation-limited. SMaRT trans-splicing enables modular replacement of defective exons, and CRISPR-based genome editing offers permanent correction, albeit with delivery and safety challenges. Incorporating knowledge of metabolic and ncRNA-based splicing regulation may enhance these approaches—for example, by integrating metabolic modulators that stabilize SR-protein phosphorylation or ncRNA-guided vector design to improve tissue specificity. Rather than competing, these modalities may be complementary, forming hybrid therapeutic strategies that integrate the short-term precision of ASOs with the long-term stability of vectorized or genome-editing systems. The field is now shifting toward precision splicing engineering, where artificial intelligence–guided optimization of splicing motifs and vector design can maximize efficiency and minimize off-target risks.

In summary, establishing a unifying model that links genetic variation, splicing dynamics, and clinical phenotype is crucial. Multi-omics integration—combining transcriptomic, proteomic, and epigenetic analyses—will be essential to uncover how splicing variants determine photoreceptor vulnerability and treatment responsiveness. Comparative studies across retinal, metabolic, and neurodegenerative disorders may further reveal shared splicing regulatory nodes with therapeutic relevance.

In conclusion, alternative splicing represents both a pathogenic hallmark and a therapeutic frontier in RP. Progress in high-resolution transcriptomics, RNA structural biology, and AI-assisted prediction promises to translate molecular insights into splicing-centered precision medicine. By bridging molecular biology, ophthalmology, and computational science, future research may ultimately enable durable and personalized interventions to preserve vision in patients with RP.

## Figures and Tables

**Table 1:** Mutations in the PRPF31 splicing factor associated with retinal pigmentosa.

Mutation Type	Nucleotide Mutation	Protein/RNA Splicing Changes	Affected Exon	Reference
Frameshift	c.849_855del	p.Pro284Ilefs*35	exon8	[54]
	c.1226_1227insA	p.T410Dfs*65	exon12	[55]
	c.357_358delAA	p.Ser119Serfs*5	exon5	[56]
	c.1115_1125del	p.Arg372Glnfs*99	exon11	[51]
	c.1035_1036insGC	p.Pro346Argfs*18	exon10	[57]
	c.1224dupG	p.Gln409Alafs*66	exon12	
	c.967_968delGA	p.E323Dfs*151	exon10	[58]
	c.327_330delCATC	p.H111Sfs*86	exon5	
	c.816_830delCTACATCTACCACAG	p.Tyr273_Ser277del	exon8	[59]
	c.1142delG	p.Gly381fs	exon11	[60]
	c.1168_1169insGATTCAGCCTGGCC	p.Glu390Glyfs*28	exon12	[61]
Splicing	c.1146+5G>T	p.Tyr359Serfs*29	Intron11	[62]
	c.855+5G>A	p.Glu333ArgAspfs*33	Intron8	[54]
	c.1146+2T>A	-	Intron11	[63]
	c.322+1G>A	p.Val80_Leu107del	Intron4	[57]
	c.527+2T>G	p.Gln177Tyrfs*10/ p.Glu141Alafs*102	Intron6	
	c.1073+5G>A	-	Intron10	[64]
	c.-9+1G>A	-	exon1	[65]
Nonsense	c.1205C>A	p.Ser402*	exon12	[66]
	c.1060C>T	p. Arg354*	exon10	[67]
	c.1015C>T	p.Glu339*	exon10	[55]
	c.220C>T	p.Gln74*	exon3	[68]
	c.1168G>T	p.Glu390*	exon 12	[65]
Missense	c.341T>A	p.Ile114Asn	exon5	[69]
	c.165G>A	p.Gly55Asp	exon2	[70]
	c.590T>C	p.Leu197Pro	exon7	[71]
	c.646G>C	p.A216Pro	exon7	[52]
	c.896G>A	p.Cys299Tyr	exon8	[72]
	c.839T>G	p.Val280Gly	exon8	[59]
Large-scale deletion	chr19:54117745–54125389 (7645 bp)	-	exon 2–8	[73]
	chr19:54110458–54130356 (19,899 bp)	-	exon 1–13	
	chr19:54109148–54133219 (24,072 bp)	-	Exon 1–14+TFPT E1–E3	
	chr19:54043540–54132981 (89,442 bp)	-	Exon 1–14+large-scale gene deletion	
	chr19:54048499–54118055 (69 kb)	-	Exon 1+5 upstream genes	[74]
	chr19:54113356–54116922	-	Promoter and 5′ untranslated region (5′ UTR)	[75]
	chr19:54113882–54116394	-		
duplication	chr19:54621606–54626745 (5.1 kb)	-	Tandem repeat of exons 2–5	[63]
	c.73_166dup	p.Asp56GlyfsTer33	exon 2	[76]

The asterisk (“*”) indicates a premature termination (stop) codon, and the adjacent numerical value specifies the position of the resultant stop codon relative to the frameshift mutation site.

**Table 2:** Splice site mutations associated with retinal pigmentosa.

Mutation Type	Gene	Nucleotide Mutation	Exon	Reference
Canonical splice donor site	*RP1*	c.615+1G>A	Intron2	[117]
	*RPGR*	c.619+1G>C	Intron6	[40]
		c.310+3A>G	Intron4	[118]
		c.619+2T>A	Intron6	
	*TTC8/BBS8*	c.1347G>C	Exon13	[119]
	*PDE6A*	c.1407+1G>C	Intron12	[120]
	*TULP1*	c.1495+1G>A	Intron14	[121]
	*RPE65*	c.1338+1G>A	Intron12	[122]
	*IMPG1*	c.1824+1G>A	Intron13	[123]
	*PDE6B*	c.1920+2T>C	Intron15	[124]
	*ARL2BP*	c.207+1G>A	Intron3	[125]
	*NRL*	c.-41_-28+23del	Exon1	[126]
Canonical splice acceptor site	*PDE6B*	c.1921-20_1921-3del	Intron15	[109]
	*RPGR*	c.470-1G>A	Intron5	[127]
		c.29-2A>T	Intron1	[118]
		c.1754-3C>G	Intron14	
	*RHO*	c.620 T > G	Exon3	[111]
	*PRPH2*	c.582-2A>T	Exon3	[128]
	*RDH11*	c.75-3C>A	Intron2	[129]
	*CDHR1*	c.1168-1G>A	Intron11	[130]
	*MAK*	c.279-2A>G	Intron3	[131]
Branch point	*BBS1*	c.592-21A>T	Intron7	[132]
Polypyrimidine tract	*RP2*	c.1073-9T>A	Intron3	[133]
Cryptic splice site	*LRAT*	c.541-15T>G	Intron2	[9]
	*PDE6B*	c.1921–9C>G	Intron15	[134]
	*RPE65*	c.1430A>G	Exon13	[110]
Non-Canonical Splice Site	*RPGR*	c.247+5G>A	Intron3	[135]
		c.154+3_154+6del	Intron2	
		c.779-5T>G	Intron7	
		c.1573-12A>G	Intron13	
		c.1415-9A>G	Intron11	[136]
	*USH2A*	c.5776G>A	Exon 28	[137]
		c.10182G>A	Exon 51	
		c.15519+2dup	Intron 71	
	*COQ5*	c.682-7T>G	Intron 5	[138]
Deep Intronic Splice Variant	*CLRN1*	c.254-643G>T	Intron1	[113]
	*RPGR*	c.1059+363G>A	Intron9	[139]
	*USH2A*	c.8682-654C>G	Intron43	[116]
	*OFD1*	c.IVS9+706A>G	Intron9	[140]

**Table 3:** Summary of therapeutic strategies targeting alternative splicing abnormalities in RP.

Therapeutic Strategy	Representative Target/Disease Model	Clinical Trial Phase	Major Findings	Key Challenges
Gene supplementation (AAV-based)	*RPE65*, *RPGR*, *PRPF31*	*Luxturna* (*RPE65*): FDA-approved (Phase IV); *RPGR*: Phase III (NCT03116113); *PRPF31*: Preclinical	Restored visual function in *RPE65*-LCA; structural and functional rescue in *RPGR* models; partial recovery in *PRPF31*-deficient RPE cells	Limited AAV cargo (~4.7 kb); inefficient for dominant-negative alleles; variable transduction efficiency
Antisense oligonucleotide (ASO) therapy	*USH2A*, *PRPF31*, *CLRN1*, *CEP290*	*QR-421a* (*USH2A*): Phase I/II (NCT03780257); *Sepofarsen or QR-110* (*CEP290*): Phase II/III for LCA10 (NCT03913143)	Corrected exon skipping or pseudoexon inclusion; restored functional protein expression and retinal function in models	Short duration, requires repeated intravitreal injection; delivery heterogeneity; mutation-specific design
U1 snRNA modification	*RHO c.936G>A*, *RPGR c.1245+3A>T*	Preclinical	Restored correct splicing and reduced exon skipping in *RHO* and *RPGR* mutations	Mutation-limited applicability; risk of overexpression or off-target binding; delivery optimization required
Spliceosome-mediated RNA trans-splicing (SMaRT)	*RHO*, *RPGR*, *ABCA4*	Preclinical	Generated hybrid mRNA restoring correct reading frame and protein localization; functional recovery in animal models	Low efficiency and reproducibility; risk of off-target chimeric transcripts; difficult stoichiometric control
Genome editing (CRISPR/Cas9, base/prime editing)	*CEP290*, *PRPF31*, *USH2A*, *RPGR*, *Pde6b*	*EDIT-101* (CEP290): Phase I/II (NCT03872479); others preclinical	Permanent correction of splice mutations; partial functional recovery in RP models	Off-target risks, low HDR efficiency in post-mitotic cells, immune response to Cas proteins, ethical concerns

## Data Availability

No new data were created or analyzed in this study.

## Abbreviations

adRP	Autosomal Dominant Retinitis Pigmentosa.
arRP	Autosomal Recessive Retinitis Pigmentosa.
ASOs	Antisense Oligonucleotides.
AAV	Adeno-Associated Virus.
ABCA4	ATP-binding cassette transporter A4.
ABE	adenine base editor.
BPS	Branch point sequence.
Brr2	Bad Response to Refrigeration 2 (also known as SNRNP200).
BBS2	Bardet–Biedl syndrome 2.
PSC	Posterior Subcapsular Cataract.
CME	Cystoid Macular Edema.
CNOT3	CCR4-NOT transcription complex subunit 3.
CCR4-NOT	carbon catabolite repression 4-negative on TATA-less.
CRISPR/Cas9	Clustered Regularly Interspaced Short Palindromic Repeats/CRISPR-Associated Protein 9.
CWC22	complexed with CEF1 protein 22.
CWC27	complexed with CEF1 protein 27.
CLRN1	Clarin 1.
CEP290	centrosomal protein 290.
CBE	cytosine base editor.
DExD/H-box	aspartate-glutamate-X-aspartate/histidine box.
DHX38	DEAH-box helicase 38.
di-snRNP	di-small nuclear ribonucleoprotein.
ESEs	exonic splicing enhancers.
ESSs	exonic splicing silencers.
eQTL	expression quantitative trait locus.
EFTUD2	elongation factor Tu GTP-binding domain containing 2.
eIF4A3	eukaryotic initiation factor 4A3.
EJC	exon junction complex.
ER	endoplasmic reticulum.
ERG	electroretinography.
eDCas9	enhanced dead Cas9.
eSpCas9	enhanced specificity Cas9.
FSCN2	fascin actin-bundling protein 2.
FDA	Food and Drug Administration.
GCL	Ganglion cell layer.
GNAT1	guanine nucleotide-binding protein alpha transducing 1.
GNAT2	guanine nucleotide-binding protein alpha transducing 2.
GPKOW	G-patch domain and KOW motifs.
GEF	guanine nucleotide exchange factor.
hnRNPs	heterogeneous nuclear ribonucleoprotein particles.
ROs	retinal organoids.
HSP70	heat shock protein 70.
hSnu114	human small nuclear U114 (alternative name for EFTUD2).
HBB	hemoglobin subunit beta.
HDR	homology-directed repair.
IPL	Inner plexiform layer.
INL	Inner nuclear layer.
ISSs	intronic splicing silencers.
IFT88	intraflagellar transport protein 88.
IFT122	intraflagellar transport protein 122.
iPSC	induced pluripotent stem cell.
Jab1/MPN	Jun activation domain-binding protein 1/Mpr1-Pad1 *N*-terminal.
LSm	like-Sm proteins.
LCA	Leber congenital amaurosis.
mRNA	messenger RNA.
MSR1	macrophage scavenger receptor 1.
MOs	morpholino oligonucleotides.
NHEJ	non-homologous end joining.
OPL	Outer plexiform layer.
ONL	Outer nuclear layer.
OMCS	Oliver–McFarlane Syndrome.
PHARC	Polyneuropathy, Hearing loss, Ataxia, Retinitis pigmentosa, and Cataract syndrome.
PCARP	Photoreceptor Ciliopathy And Retinal Pigmentation.
PPT	Polypyrimidine tract.
pre-mRNA	precursor messenger RNA.
PTBP1	polypyrimidine tract-binding protein 1.
PRPF3	pre-mRNA processing factor 3.
PRPF4	pre-mRNA processing factor 4.
PRPF6	pre-mRNA processing factor 6.
PRPF8	pre-mRNA processing factor 8.
PRPF31	pre-mRNA processing factor 31.
PAP-1	polyadenylation specificity factor 1.
PPIase	peptidyl-prolyl cis-trans isomerase.
PDE6B	Phosphodiesterase 6B.
PTMs	pre-mRNA trans-splicing molecules.
RPE	Retinal pigment epithelium.
RP	Retinitis Pigmentosa.
RBM20	RNA-binding motif protein 20.
RPGR	retinitis pigmentosa GTPase regulator.
RPCs	retinal progenitor cells.
RHO	rhodopsin.
RPGRIP1	retinitis pigmentosa GTPase regulator interacting protein 1.
RPE65	Retinal Pigment Epithelium 65.
rAAV	recombinant adeno-associated virus.
RAB37	Ras-related protein 37.
REVeRT	mRNA trans-splicing recombinant technology.
PPIH	peptidyl prolyl isomerase H.
Sm	Smith antigens.
snRNA	small nuclear RNA.
snRNPs	small nuclear ribonucleoprotein complexes.
SF1	Splicing Factor 1.
SR	serine/arginine-rich.
SNRNP200	small nuclear ribonucleoprotein 200.
siRNA	small interfering RNA.
SC35	serine/arginine-rich splicing factor 2.
SaCas9	Staphylococcus aureus Cas9.
sgRNA	single guide RNA.
SMaRT	spliceosome-mediated RNA trans-splicing.
tri-snRNP	U4/U6.U5 tri-snRNP complex.
TPR	tetratricopeptide repeat.
TTLL3	tubulin tyrosine ligase like 3.
U snRNAs	uridine-rich small nuclear RNAs.
U2AF1	U2 Auxiliary Factor 35 kDa subunit.
U2AF2	U2 Auxiliary Factor 65 kDa subunit.
ULK1	unc-51 like autophagy activating kinase 1.
USH2A	Usherin 2A.
U1_asRNA	U1 antisense small nuclear RNA.
WD40	WD repeat domain 40.
XLRP	X-Linked Retinitis Pigmentosa.
5′ ss	5′ splice site.
3′ ss	3′ splice site.
